# Platelet-Mediated Protection of Cancer Cells From Immune Surveillance – Possible Implications for Cancer Immunotherapy

**DOI:** 10.3389/fimmu.2021.640578

**Published:** 2021-03-10

**Authors:** Laurent Schmied, Petter Höglund, Stephan Meinke

**Affiliations:** Department of Medicine Huddinge, Center for Hematology and Regenerative Medicine, Karolinska Institutet, Huddinge, Sweden

**Keywords:** NK cells, platelets, immunosuppressive, tumor microenvironment, antitumor immunity, metastasis, immunotherapy, cytotoxicity

## Abstract

The growing insights in the complex interactions between metastatic cancer-cells and platelets have revealed that platelet tumor cell interactions in the blood stream are an important factor supporting tumor metastasis. An increased coagulability of platelets facilitates the vascular evasion and establishment of solid tumor metastasis. Furthermore, platelets can support an immunosuppressive tumor microenvironment or shield tumor cells directly from engagement of cytotoxic lymphocytes as e.g., natural killer (NK) cells. Platelets are both in the tumor microenvironment and systemically the quantitatively most important source of TGF-β, which is a key cytokine for immunosuppression in the tumor microenvironment. If similar platelet-tumor interactions are of physiological relevance in hematological malignancies remains less well-studied. This might be important, as T- and NK cell mediated graft vs. leukemia effects (GvL) are well-documented and malignant hematological cells have a high exposure to platelets compared to solid tumors. As NK cell-based immunotherapies gain increasing attention as a therapeutic option for patients suffering from hematological and other malignancies, we review the known interactions between platelets and NK cells in the solid tumor setting and discuss how these could also apply to hematological cancers. We furthermore explore the possible implications for NK cell therapy in patients with solid tumors and patients who depend on frequent platelet transfusions. As platelets have a protective and supportive effect on cancer cells, the impact of platelet transfusion on immunotherapy and the combination of immunotherapy with platelet inhibitors needs to be evaluated.

## Introduction

Natural killer (NK) cells represent the largest fraction of innate lymphocytes, accounting for 10–15% of all peripheral lymphocytes in humans (1, 2), The physiological importance of NK cells is commonly ascribed to their capability to form early responses against viral infections and malignant cells (3). The main effector functions of NK cells encompass elimination of cells identified as targets, along with the secretion of proinflammatory cytokines, which can attract further immune cells and thereby promote the formation of an adaptive immune response (4). NK cell activation is regulated by the integration of signals from an array of different germline-encoded activating and inhibitory receptors (5). Activating receptors on NK cells bind many stress-induced ligands as for example MICA and MICB, which are recognized by NKG2D on NK cells (6). The main inhibitory receptors are the killer cell immunoglobulin like receptors (KIR) which bind to HLA class I and NKG2A which binds to HLA-E, thereby allowing NK cells to kill virally infected or transformed cells that escape T cell-mediated immunosurveillance by down-regulation of HLA (3, 7, 8). This unique capability of immediate cytotoxicity toward malignant and virally infected cells makes NK cells attractive for antitumor therapy approaches (9). Especially since a clinically relevant antitumor effect has been described before, when a NK cell-mediated graft vs. leukemia effect (GvL) was found after haploidentical hematopoietic stem cell transplantation (HSCT), in case of HLA mismatch when NK cell tolerance is broken (10, 11). Currently, different anti-tumor therapies rely on tumor cell lysis through cytotoxic lymphocytes, mainly NK- and T cells. This includes beside NK- and T-cell-mediated GvL after HSCT (10, 12) the therapies inhibiting immune checkpoints (13). Moreover, the success of monoclonal antibody therapies including e.g., Rituximab depends on NK cell-mediated antibody dependent cytotoxicity (ADCC) as a main effector function (14). Lastly, there are many studies evaluating the therapeutic value of expanded NK cells or therapies with engineered NK cells or T cells (15–18).

Platelets have a well-established and important role in hemostasis and wound healing, but it has become clear that they also function as immune cells (19). In the context of cancer, they have been shown to support various steps of tumor expansion, including local growth, migration in and out of the blood stream and metastasis establishment. During several of those steps platelets are important for evasion of the immune system (20). The protection from immunosurveillance can be the result of a direct or indirect inhibition of tumor cell engagement by cytotoxic lymphocytes. Here, we review the different ways how platelets can support cancer cells to avoid or disarm lymphocyte cytotoxicity focusing on NK cells. We furthermore explore the possible implications of NK cell platelet-interactions on NK cell therapy and platelet transfusions.

## Platelet-Mediated Immune Escape Mechanisms in the Tumor Environment

High platelet counts were identified as a risk factor associated with adverse outcome in numerous different tumor entities including lung cancer, breast cancer, ovarian cancer, gastric cancer, pancreas carcinoma, hepato-cellular carcinoma, colon carcinoma, renal cell carcinoma or glioblastoma to name just a few (21–28). Based on these robust data, and studies showing that low lymphocyte counts correlate with shorter survival time (29), the ratio between lymphocytes and platelets has been investigated and identified as a predictive marker for the disease outcome with low platelet-lymphocyte ratios (PLR) favoring a beneficial course of the disease (30–32). Of note, a meta-analysis including 1,340 cancer patients that were treated with an immune checkpoint inhibitor, showed a clear advantage of patients with a low PLR (33). In sum, these observational studies gave rise to the question if platelet count and PLR are mere surrogate markers indicating strong systemic inflammatory response, reflecting advanced progression, or if clinically relevant interactions between platelets and lymphocytes can influence the disease outcome by themselves. Notably, these two explanations are not mutually exclusive. The understanding of how platelets can interfere with the function of different lymphocyte subsets has significantly grown and different mechanisms were uncovered. It was shown that platelets protect tumor cells from different cytotoxic lymphocytes including NK cells and effector T cells. The protective mechanisms from lysis by NK cells can be divided in those resulting from direct interaction including cell contact and cytokine interaction and those involving further cell types (34).

**Figure 1:**
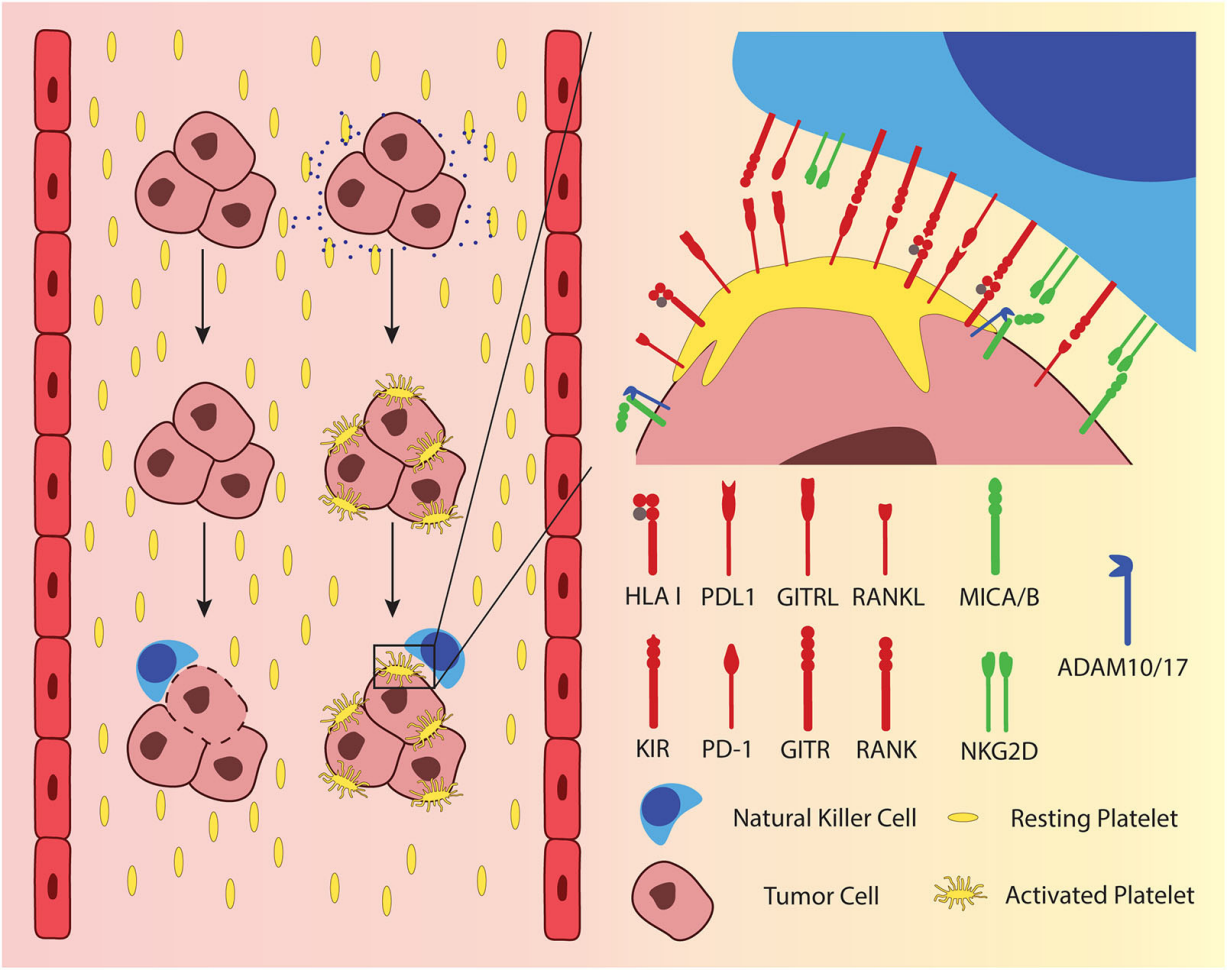
Metastasizing tumor cells exploit platelets as physical and immunological shielding from NK cells in peripheral blood. Platelets are activated upon encountering tumor cells, which release adenosine diphosphate (ADP) and tissue factor (TF), depicted as blue dots. Activated platelets adhere to the tumor cells surface, providing a physical and an immunological shield by presenting ligands to inhibitory NK cell receptors. Moreover, activated platelets exchange surface receptors with tumor cells. Several ligands to inhibitory NK cell receptors have been shown to be passed over from platelets to tumor cells, including HLA class I, glucocorticoid-induced TNF-related protein (GITR) ligand, receptor activator of NFκB (RANK) ligand and PD-L1. The figure furthermore delineates the shedding of MICA and MICB, which are ligands to the activating NK cell receptor NKG2D.

When a single cell or micro metastasis, consisting of a few cells enters the blood stream, it is at the same time leaving the immunosuppressive, protective environment of the tumor. It becomes vulnerable and is more exposed to potential recognition and elimination by the immune system. Metastatic tumor cells that enter the blood can activate platelets by tissue factor (TF)-mediated thrombin generation and the release of ADP or Thromboxane A2 (TXA2) (35, 36). The activated platelets can attach to the cancer cells via integrins, fibrin, and P-selectin, forming a layer of platelets, hiding the malignant cell from cellular components of the immune system (37) (Figure 1, left). This “cloaking” of cancer cells with platelets protects them from NK cell-mediated lysis as it was first described in mouse models of metastatic cancers (38, 39). Initially, it was hypothesized that the platelets would simply physically shield the cancer cells from direct interaction with the NK cells. More recent research on solid tumor-derived cancer cells showed that there are several more specific mechanisms by which the adherent platelets inhibit activation of NK cells. Adherent platelets can transfer their ligands for inhibitory NK cell receptors to the cancer cell surface, namely HLA class I (40), glucocorticoid-induced TNF-related protein (GITR) ligand (41), and the receptor activator of NFκB (RANK) ligand (42) (Figure 1, right). While KIRs, the receptors for HLA class I, are constitutively expressed on the majority of circulating NK cells, the latter two inhibitory receptors are only up-regulated under certain circumstances. GITR is expressed at low levels in resting NK cells from healthy donors but is up-regulated after activation through IL-2 or IL-15 (43). RANK expression is also absent on resting NK cells from healthy donors but is found on NK cells from patients with AML (44), breast cancer, and colon cancer (42). A recent report suggested that platelet-derived PD-L1 could protect PD-L1-negative solid tumors from elimination by T cells (45), a mechanism that extends also to NK cells (46). In addition, adherent platelets can promote the shedding of the NKG2D ligands MICA and MICB from the cancer cell surface through ADAM10/17-mediated cleavage (47, 48) (Figure 1, right). It has also been shown that platelet-coated tumor cells have less detectable CD112 and CD155 on their surface, the ligands to the activating NK cell receptor DNAM-1 (48). If the mechanism of this reduction involves the interaction of platelet DNAM-1 with its ligands on the cancer cells was not investigated.

The “cloaking” of cancer cells by platelets has mostly been studied in the context of solid tumor metastasis. However, it is very likely to play a role in hematological malignancies, too. Platelets attach to leukocytes in the circulation of healthy donors (49) and have been shown to adhere to the erythroleukemia cell line K562 (48), as well as primary acute myelogenous leukemia (AML) cells *in vitro* (50). Shedding of NKG2D ligands was observed when platelets attached to K562 cells, similar to cell lines derived from solid tumors (48). Hematological malignancies are often accompanied by thrombocytopenia or platelet dysfunction, a fact that makes platelets appear less likely to play a significant role in the immune evasion of these cancer types, which can explain why the interactions of platelets with hematological cancer cells have been less well-studied. However, in a small study, platelets were found attached to circulating AML blast in three out of eight patients (50) and a study with cryopreserved material from over 1,000 AML patients found platelets adherent to AML blasts in about one third of the cases (51). It appears therefore plausible that leukemia cells may benefit from adhering platelets in a similar way as metastasizing cells from solid tumors do. In consequence, those leukemia cells might be more difficult to target by host immunity, monoclonal antibodies or cellular immunotherapies.

**Figure 2:**
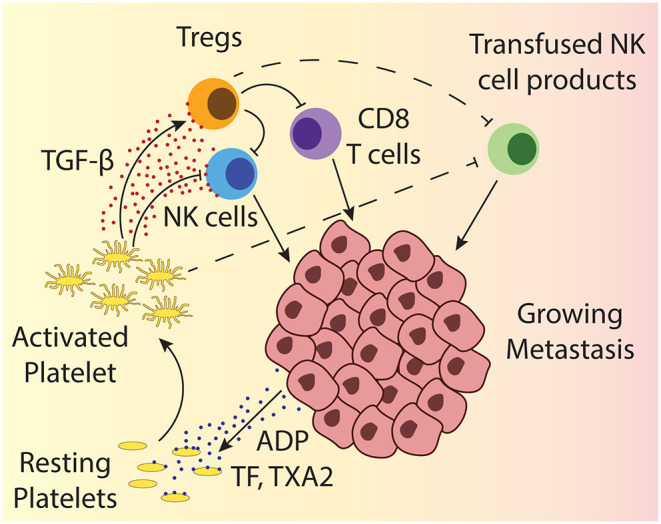
Depicted is a growing metastasis, highlighting the interactions between platelets and different lymphocytes. Tumor cells can activate platelets through release of ADP and TF. ADP directly activates platelets through engagement of the ADP receptor, while TF activates platelets together with other coagulation factors. Platelet activation results in the release of the dense granules and α-granules that contain amongst others more ADP, different clotting factors, growth factors, and TGF-β, which can impact different tumor infiltrating lymphocytes. The impact of TGF-β on T cells and NK cells is indicated by solid line arrows and the suspected impact on transfused cellular therapy products is shown by dashed line arrows.

Beside the direct contact-dependent tumor cloaking, platelets can support tumor growth and metastasis through the secretion of various factors (Figure 2). The granules released by platelets upon activation contain amongst others several factors of the coagulation cascade, growth factors and cytokines including TGF-β (52). Platelets are, in fact, the main source of TGF-β in the human body, both systemically and also specifically in the tumor microenvironment (53–56). TGF-β figures among the most extensively investigated immunosuppressive cytokines in the tumor microenvironment and it has been demonstrated to exert deleterious effects by affecting different lymphocytes. TGF-β inhibits the differentiation of T cells into cytotoxic T cells and raises the number of regulatory T cells (Tregs) (57–59). Tregs in turn can inhibit effector T cells and NK cells. TGF-β also exerts direct impact on NK cells: It has been shown that TGF-β impairs the lytic activity as well as IFN-γ production (60, 61). The mechanisms how TGF-β reduces NK cell activity encompass downregulation of activating receptors on NK cells as e.g., NKp30 or NKG2D, resulting in decreased capability to kill target cells with the respective ligands (55, 62) and interference with IFN-γ transcription (63). Importantly and beside the fact that platelets are the main source of TGF-β it has been specifically proven that platelet-derived TGF-β can impair cytokine production and degranulation of NK cells (55, 64). Taken together, the TGF-β provided by platelets can restrain cellular immune responses in solid tumors, as well as in the bone marrow microenvironment of hematological malignancies and thus support the survival of cancer cells.

## Discussion

Beside the physiological role of NK cells in the control of transformed or virally infected cells, they are also a cornerstone of monoclonal antibody therapies as a mediator of ADCC (14, 65). In addition, NK cells exert, beside T cells, graft vs. tumor effects in allogeneic HSCT settings (10, 15) and various cellular therapy approaches based on *ex vivo* activated or expanded NK cells are pursued (18). In all these settings, it is a common goal to maximize cytotoxicity to achieve tumor eradication. While it remains so far elusive if and to which extent the killing capacity of cellular therapy products are reduced by platelets in the tumor microenvironment, we extrapolate that the infused cellular therapies are likewise inhibited by platelets. Therefore, it is necessary to assess the impact of tumor PLT interactions on cytotoxicity of cellular therapy products. Similar considerations apply to further immunotherapies, that rely on tumor cell lysis mediated by other cytotoxic lymphocytes, especially the inhibition of checkpoints for T cell activation e.g., by anti-CTLA4, anti-PD1 or anti-PDL1 antibodies, as well as therapies with engineered T cells. An increasing number of different immunotherapies which rely on NK cell and other lymphocyte-mediated effector functions find already broad application or have the perspective of doing so (18, 66). With a growing understanding of the adverse effects of tumor cell-platelet interactions and the consequences on various lymphocytes, new questions arise. Starting from practical questions; a large portion of patients with hematological malignancies e.g., receive frequent infusions of platelet products (67). Regarding the immunosuppressive potential of platelets described above, an optimization of the transfusion management might be worth considering if the patient is in need of both platelets and cellular therapy at the same time, especially as platelet storage increases the available TGF-β in the transfusion unit (64). Beyond this, it is highly desirable to find a generally applicable approach to prevent the adverse interactions between platelets and NK cells. This may also be beneficial for cancer patients regardless if they receive immunotherapy. While it was previously not the scope to target interactions between platelets and NK cells specifically, different approaches were evaluated either aiming to reduce the number of platelets in the tumor microenvironment or to prevent the activation of platelets or their interaction with tumor cells: It was shown that a specific inhibition of tumor-associated platelets by directing the platelet inhibitor ticagrelor to tumor-associated platelets, using a tumor homing liposomal nanoparticle strongly reduced lung metastases in a mammary carcinoma mouse model (68). Another recent study investigated the possibility of targeting cancer cell TF expression with nanoparticle-mediated delivery of siRNA to the site of metastasis in a breast cancer mouse model. This led to a tumor specific silencing of tissue factor, which in turn resulted in reduced platelet adhesion and ultimately in lower numbers of lung metastases (69). Similar observations were made studying the impact of systemic ticagrelor treatment in a breast cancer mouse model (70). However, while NK cell-mediated killing of K562 *in vitro* was inhibited in the presence of platelets, no difference was observed if the platelets were pre-treated with ticagrelor or not (71). A very recent study in mice showed reduced metastasis and a reversal of TGF-β mediated immunosuppression upon delivery of NO-releasing nanoparticles. The NO released, inhibited platelet activation and therefore TGF-β release specifically in the tumor microenvironment (72). A limitation that all approaches targeting the platelet activation share, is that the desired interruption of tumor platelet interaction with tumor cells comes with the risk of reduced coagulation function of platelets. Inhibiting platelet activation is therefore likely unsuitable for thrombocytopenic patients but might be a promising therapy for patients with normal thrombocyte counts, especially when the delivery of the platelet inhibitor can be targeted to the tumor microenvironment and unspecific coagulation inhibition can be further reduced.

## Conclusion

As platelets can protect cancer cells from cytotoxic lymphocytes by various mechanisms, it is very suggestive that they have an impact on the efficiency of cancer immunotherapies. So far, most studies have investigated either immunotherapy or inhibition of platelet activation, but not the combination of both. Future research will show if targeting the protective interaction of platelets with tumor cells can improve the efficiency of NK cell therapy in solid tumors where results so far were less promising than in immunotherapy of hematological malignancies. On the other hand, in hematological malignancies that are often accompanied by thrombocytopenia, it is less likely that inhibition of platelet activation would be beneficial for the patients. Here, however, the effects of platelets on NK cell cytotoxicity should be considered in the scheduling of platelet transfusions and treatment.

## Author Contributions

LS, PH, and SM wrote the manuscript. All authors contributed to the article and approved the submitted version.

## Conflict of Interest

The authors declare that the research was conducted in the absence of any commercial or financial relationships that could be construed as a potential conflict of interest.
